# Racial Differences in Patient-Reported Symptoms and Adherence to Adjuvant Endocrine Therapy Among Women With Early-Stage, Hormone Receptor–Positive Breast Cancer

**DOI:** 10.1001/jamanetworkopen.2022.25485

**Published:** 2022-08-10

**Authors:** Xin Hu, Mark S. Walker, Edward Stepanski, Cameron M. Kaplan, Michelle Y. Martin, Gregory A. Vidal, Lee S. Schwartzberg, Ilana Graetz

**Affiliations:** 1 Department of Health Policy and Management, Rollins School of Public Health, Emory University, Atlanta, Georgia; 2 ConcertAI, Boston, Massachusetts; 3 Gehr Family Center for Health Systems Science and Innovation, Keck School of Medicine, University of Southern California, Los Angeles; 4 Center for Innovation in Health Equity Research, Department of Preventive Medicine, College of Medicine, University of Tennessee Health Science Center, Memphis; 5 West Cancer Center and Research Institute, Germantown, Tennessee; 6 Division of Hematology and Oncology, College of Medicine, University of Tennessee Health Science Center, Memphis; 7 Renown Institute for Cancer, Reno, Nevada

## Abstract

**Importance:**

Adjuvant endocrine therapy (AET) reduces breast cancer recurrence, but symptom burden is a key barrier to adherence. Black women have lower AET adherence and worse health outcomes than White women.

**Objective:**

To investigate the association between symptom burden and AET adherence differences by race.

**Design, Setting, and Participants:**

A retrospective cohort study using electronic health records with patient-reported data from a large cancer center in the US. Patients included Black and White women initiating AET therapy for early-stage breast cancer from August 2007 to December 2015 who were followed for 1 year from AET initiation. Sixty symptoms classified into 7 physical and 2 psychological symptom clusters were evaluated. For each cluster, the number of symptoms with moderate severity at baseline, and symptoms with 3-point or greater increases during AET were counted. Adherence was measured as the proportion of days covered by AET during the first-year follow-up. Multivariable regressions for patients’ adherence adjusting for race, symptom measures, sociodemographic characteristics, and clinical characteristics were conducted. Kitagawa-Blinder-Oaxaca decomposition was used to quantify racial differences in adherence explained by symptoms and patient characteristics. Analyses were conducted from July 2021 to January 2022.

**Exposures:**

Physical and psychological symptoms at baseline and changes during AET.

**Results:**

Among 559 patients (168 [30.1%] Black and 391 [69.9%] White; mean [SD] age 65.5 [12.1] years), Black women received diagnoses younger (mean [SD] age at diagnosis, 58.7 [13.7] vs 68.5 [10.0] years old) than White women, with more advanced stages (30 Black participants [17.9%] vs 31 White participants [7.9%] had stage III disease at diagnosis), and lived in areas with fewer adults attaining high school education (mean [SD], 78.8% [7.8%] vs 84.0% [9.3%]). AET adherence in the first year was 78.8% for Black and 82.3% for White women. Black women reported higher severity in most symptom clusters than White women. Neuropsychological, vasomotor, musculoskeletal, cardiorespiratory, distress, and despair symptoms at baseline and increases during the follow-up were associated with 1.2 to 2.6 percentage points decreases in adherence, which corresponds to 4 to 9 missed days receiving AET in the first year. After adjusting for psychological symptoms, being Black was associated with 6.5 percentage points higher adherence than being White.

**Conclusions and Relevance:**

In this cohort study, severe symptoms were associated with lower AET adherence. Black women had lower adherence rates that were explained by their higher symptom burden and baseline characteristics. These findings suggest that better symptom management with a focus on psychological symptoms could improve AET adherence and reduce racial disparities in cancer outcomes.

## Key Points

**Question:**

Are racial differences in symptom burden during the first year of adjuvant endocrine therapy (AET) associated with differences in adherence?

**Findings:**

In this cohort study of 559 patients from a large cancer center, Black women with early-stage breast cancer experienced higher physical and psychological symptom burden during the first year of AET. Each additional moderate severity symptom was associated with 4 to 9 missed days receiving AET.

**Meaning:**

These findings suggest that better symptom management could improve AET adherence and reduce racial disparities in adherence and cancer outcomes.

## Teaser

This cohort study evaluates the differences in patient-reported symptoms and adherence to adjuvant endocrine therapy by race among women with early-stage breast cancer.

## Introduction

Adjuvant endocrine therapy (AET) has substantial clinical benefits, including a reduction in breast cancer recurrence by 20% to 30% among women with hormone receptor–positive breast cancer.^1,2^ However, AET adherence remains a concern in the US,^3,4^ and Black women are more likely to have lower adherence compared with White women.^5,6^ Both physical and psychological symptoms are key barriers to AET adherence.^7,8,9,10^

Black women experience a higher symptom burden and more frequently face undertreatment of symptoms, such as pain.^11,12,13,14,15,16^ Differences in socioeconomic status, higher rates of exposure to environmental risk factors, lower access to health care, and explicit and implicit clinician bias driven by centuries of racism, oppression, and disinvestment, rather than biological differences, contribute to higher symptom burden among Black women.^11,16,17,18,19,20,21,22^ Race is a social construct,^23^ and observed differences in health experiences and outcomes by marginalized groups must be understood in the broader context of structural racism and other factors that differentially place minorities at increased risks for high symptom burden, enduring disparities in treatment adherence, and breast cancer outcomes.^24,25^

Prior studies^26,27,28^ comparing symptom burden differences by race were unable to adjust for differences in baseline symptom severity or evaluate symptom burden changes during AET treatment. One recent study^29^ assessed AET-related symptoms collected from a cross-sectional patient survey and found that after adjusting for symptom severity, Black women were still less likely to adhere to AET than White women. Evidence of symptom burden changes after starting AET and how differences in symptom burden changes are associated with differences in adherence by race is lacking. This cohort study used longitudinal patient-reported symptom data to understand racial differences in symptom burden during AET and to quantify differences in AET adherence explained by differences in symptom burden and other patient characteristics among women with early-stage hormone receptor–positive breast cancer.

## Methods

### Data and Analytical Sample

We used electronic health records linked with patient-reported symptom data to identify a cohort of women with early-stage, hormone receptor–positive breast cancer receiving care from the West Cancer Center and Research Institute (WCCRI). WCCRI is a large multidisciplinary cancer center consisting of 9 clinics and more than 70 physicians serving 60% of all patients in the tri-state area of west Tennessee, north Mississippi, and east Arkansas. Patient-reported outcomes were routinely collected during patient visits using the Patient Care Monitor (PCM) tablet platform (ConcertAI).^30^ We obtained data from the Tennessee cancer registry, and Medicare and Medicaid claims to retrieve patients’ cancer history, cancer treatments, and other health care services received outside WCCRI. The 2011 to 2015 American Community Survey 5-year estimates file was used for neighborhood-level sociodemographic information. This study was approved by the University of Tennessee Health Science Center institutional review board and follows the Strengthening the Reporting of Observational Studies in Epidemiology (STROBE) reporting guidelinefor cohort studies. The University of Tennessee Health Science Center waived the requirement for written informed consent, in accordance with 45 CFR §46.

The study sample was restricted to patients who had any AET drug claims (eTable 1 in the Supplement) between August 2007 to December 2015, no missing clinical characteristics needed for the analysis, and identified as White or Black race only because of limited sample sizes of other racial groups. We required Medicare or Medicaid coverage from 2 months before to 1 year after patients’ first AET drug claim (ie, AET initiation), no missing zip code to link to neighborhood-level characteristics, at least 1 PCM report within 2 months before AET initiation, and 1 within the first year of AET initiation to measure symptom changes during AET (eFigure in the Supplement). Eligible patients were followed for 1 year after AET initiation.

### Patient-Reported Symptoms and Adherence

The PCM Survey has been previously validated and used in studies of symptom burden among patients with cancer.^30,31^ It screens for symptoms commonly observed in patients with cancer, including physical and psychological symptoms and physical functioning. Patients rated the severity of each symptom in the past week on a 0 to 10 scale, from 0 (not a problem) up to 10 (as bad as possible) (eTable 2 in the Supplement). We evaluated all 60 symptom items collected from the PCM survey, and classified them into 7 physical and 2 psychological clusters according to previous literature and the clinical expertise from oncologists in our study team.^29^ These 9 clusters are gastrointestinal symptoms, gynecological symptoms, neuropsychological symptoms, vasomotor symptoms, musculoskeletal symptoms, integumentary symptoms, cardiorespiratory symptoms, distress symptoms, and despair symptoms. A detailed list of individual symptoms in each cluster is provided in eTable 3 in the Supplement.

We measured patients’ baseline severity of each symptom item from the closest PCM report within 2 months before AET initiation, and the changes from the baseline report to the highest severity reported during the 1-year follow-up period. We focused on the change from baseline to highest severity to capture the full range in symptom burden for women starting AET. We then counted the number of symptoms with moderate severity (ie, ≥3 points on a 0-10 scale), and the number of symptoms with a 3-point or greater increase during therapy within each cluster. The 3-point cutoff was chosen because it represents a clinically meaningful symptom burden change.^32^

We measured adherence as the proportion of days covered (PDC) by AET drugs during the first year of therapy.^33^ PDC is a widely used claim-based adherence measure that has been validated in previous research.^33,34,35^ Total days of supply were calculated from all AET claims that occurred within 1 year of AET initiation. If the last AET prescription coverage extended beyond 1 year, we replaced the final day of medication coverage with the last date of the 1-year follow-up period, to ensure a 0% to 100% continuous measure of adherence.

### Covariates

To account for other factors that may affect patients’ ability to fill their prescription, we adjusted for characteristics according to Andersen’s behavioral model of health service use.^36^ Sociodemographic factors including age at diagnosis and state of residence were available from the electronic health record. Insurance (Medicare only, Medicaid, or dual eligible) was measured using Medicaid and Medicare enrollment files. Neighborhood-level median household income and percent of adults with a high school degree were linked from the American Community Survey using zip codes or county codes where zip code–level data were not available. Clinical characteristics that may contribute to symptom burden were extracted from the electronic health record and claims, including cancer stage at diagnosis,^37,38^ history of chemotherapy,^39,40,41^ and first AET drug type.^42,43^

### Statistical Analyses

Baseline characteristics were described using mean and SD for continuous variables, and frequency as well as percentage for categorical variables. We presented the mean symptom severity at baseline and changes during AET therapy for each individual symptom and compared the count of moderate symptoms at baseline and the count of symptoms with a 3-point or greater increase between 2 racial groups. Multivariable linear regression was used to assess the association of patients’ symptom burden at baseline and during AET with adherence, adjusting for race, sociodemographic characteristics, and clinical characteristics. Because of multicollinearity concerns among these symptom clusters, we included 1 symptom cluster in the model at a time. Variance inflation factors and correlation tests were estimated for covariates in each model, which showed a low level of multicollinearity.

To understand how differences in symptom burden between Black and White patients explain the differences in their adherence to AET, we used the Kitagawa-Blinder-Oaxaca method to decompose the net differences by race in adherence into explained differences by observable covariates and unexplained differences. This method is widely used to study gender or race-related disparities.^7,44,45,46,47^ It builds upon ordinary least square models run among White and Black patients separately. The explained difference was calculated as the sum of the differences in White and Black patient characteristics (ie, values of the covariates) multiplied by coefficients from the White patients’ ordinary least squares model. A positive explained difference means that if Black patients had shared the same characteristics as White patients, their adherence would be higher, and vice versa. The unexplained difference reflects the difference in coefficients from White and Black patients’ ordinary least squares model, when applied to the average Black patients’ characteristics. It can be interpreted as the difference in adherence that would remain if Black and White patients had the same baseline characteristics. Similar to the multivariable regression analysis, each decomposition model included 1 symptom cluster.

SAS statistical software version 9.4 (SAS Institute) and Stata statistical software version 13 (StataCorp) were used for our analyses. Statistical significance was determined using 2-sided tests with an α of .05. Analyses were conducted from July 2021 to January 2022.

## Results

A total of 559 participants were included in our analysis. There were 168 Black women (30.1%) and 391 White women (69.9%), with a mean (SD) age of 65.5 (12.1) years (Table 1). Compared with White women, Black women received diagnoses at a younger age (mean [SD] 58.7 [13.7] vs 68.5 [10.0] years old; *t*_246.66_ = −8.31; *P* < .001) and with a more advanced stage (stage III, 30 patients [17.9%] vs 31 patients [7.9%]; χ^2^_2_ = 21.3493; *P* < .001). They were more likely to have received chemotherapy (93 patients [55.4%] vs 125 patients [31.9%]; χ^2^_2_ = 27.4011; *P* < .001), lived in poorer neighborhoods (neighborhood-level median household income <$35 000: 107 patients [63.7%] vs 108 patients [27.6%]; χ^2^_3_ = 70.7095; *P* < .001), neighborhoods with fewer adults attaining high school education (mean [SD] 78.8% [7.8] vs 84.0% [9.3]; *t*_373.36_ = −6.73; *P* < .001). In addition to the baseline PCM, a mean (SD) of 3.5 (2.7) PCM surveys were collected during patients’ first year of AET. Black women completed more surveys than White women (mean [SD] 3.9 [3.0) vs 3.3 [2.5]; *t*_267.24_ = 2.14; *P* = .03). Sample characteristics for the larger cohorts without requiring continuous insurance coverage or having PCM report showed similar distribution (eTable 6 in the Supplement).

**Table 1.** Patient Characteristics by Race

Characteristic	Patients, No. (%)		
	Total (N = 559)	Black (n = 168)	White (n = 391)
Age at diagnosis, mean (SD), y	65.5 (12.1)	58.7 (13.7)	68.5 (10.0)
Cancer stage at diagnosis			
I	280 (50.1)	62 (36.9)	218 (55.8)
II	218 (39.0)	76 (45.2)	142 (36.3)
III	61 (10.9)	30 (17.9)	31 (7.9)
Neighborhood level high school education, mean (SD)	82.4 (9.2)	78.8 (7.8)	84.0 (9.3)
Neighborhood level annual household income group, $			
<35 000	215 (38.5)	107 (63.7)	108 (27.6)
35 000-44 999	98 (17.5)	25 (14.9)	73 (18.7)
45 000-54 999	82 (14.7)	17 (10.1)	65 (16.6)
≥55 000	164 (29.3)	19 (11.3)	145 (37.1)
State^a^			
Tennessee	359 (64.2)	139 (82.7)	220 (56.3)
Mississippi	165 (29.5)	23 (13.7)	142 (36.3)
History of chemotherapy			
No chemotherapy identified	341 (61.0)	75 (44.6)	266 (68.0)
Chemotherapy within 180 d before AET/after AET initiation	101 (18.1)	41 (24.4)	60 (15.3)
Chemotherapy beyond 180 d before AET	117 (20.9)	52 (31.0)	65 (16.6)
Insurance type			
Medicare only	425 (76.0)	75 (44.6)	350 (89.5)
Medicaid or dual	134 (24.0)	93 (55.4)	41 (10.5)
First AET drug type			
Tamoxifen	54 (32.1)	61 (15.6)	115 (20.6)
Anastrozole	66 (39.3)	225 (57.5)	291 (52.1)
Exemestane	7 (4.2)	46 (11.8)	53 (9.5)
Letrozole	41 (24.4)	59 (15.1)	100 (17.9)
All AET drug types during first year			
AI only^b^	97 (57.7)	276 (70.6)	373 (66.7)
Tamoxifen only	36 (21.4)	50 (12.8)	86 (15.4)
AI and tamoxifen^b^	35 (20.8)	65 (16.6)	100 (17.9)
Adherence measures			
Days covered by AET, mean (SD), %	81.2 (27.0)	78.8 (27.2)	82.3 (26.9)
PCM report characteristics			
Time from baseline PCM to AET initiation, mean (SD), d	8.8 (14.3)	8.9 (14.5)	8.7 (14.2)
PCM reports within 1 y after AET initiation, mean (SD), No.	3.5 (2.7)	3.9 (3.0)	3.3 (2.5)

Abbreviations: AET, adjuvant endocrine therapy; AI, aromatase inhibitors; PCM, Patient Care Monitor.

^a^ A total of 23 patients resided in the state of Arkansas and 12 patients resided in states other than Tennessee, Mississippi, or Arkansas.

^b^ AIs include anastrozole, exemestane, and letrozole.

### Symptom Changes During AET Overall and By Race

**Figure 1.**
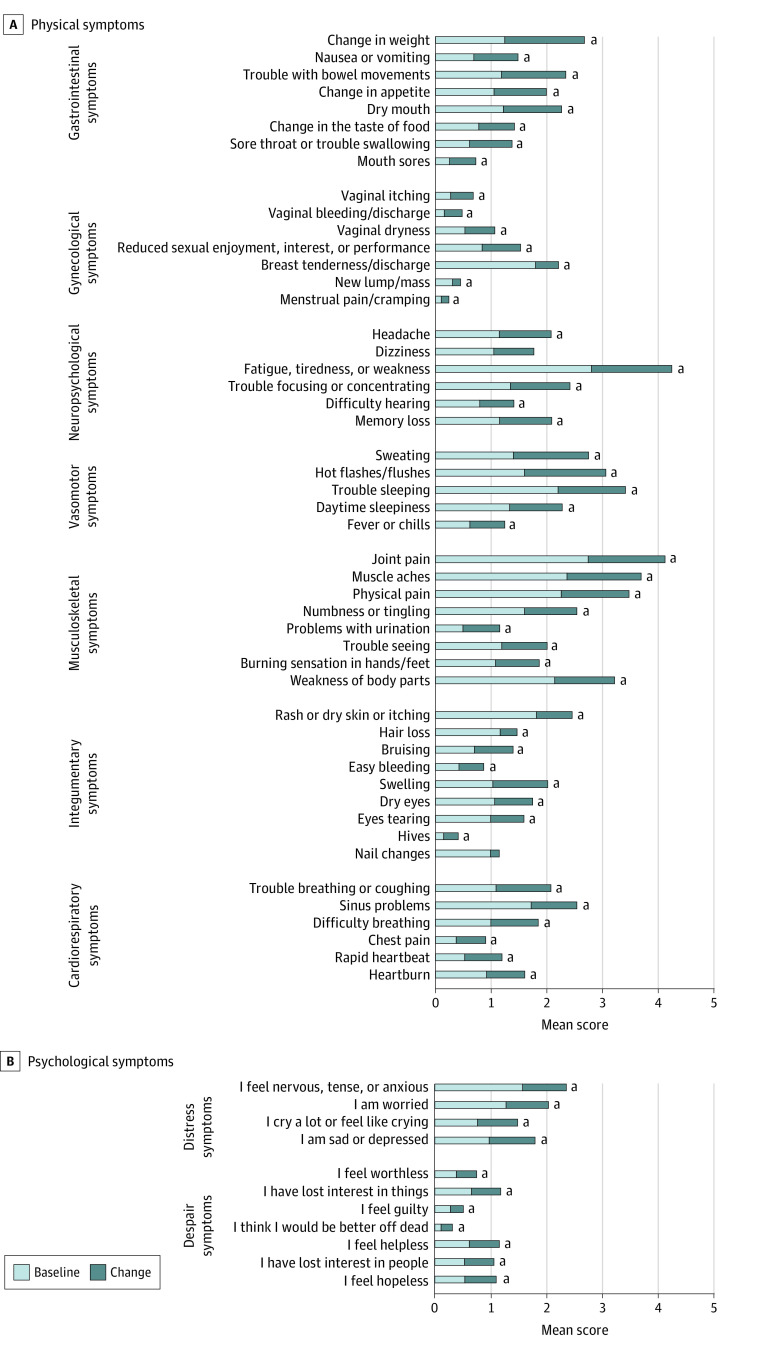
Mean Score at Baseline and Changes During 1-Year Follow-up for Individual Symptom Items Individual symptom items are scored on a 0 to 10 scale, where 0 indicates not a problem and 10 indicates as bad as possible. Baseline means were reported within 2 months before starting adjuvant endocrine therapy and changes from the baseline were determined by the highest severity symptoms reported during the 1-year follow-up period. ^a^Denotes changes from baseline significantly different from 0 (*P* < .05).

Figure 1 presents the mean baseline severity and mean changes during the first year of AET for each individual symptom. Although many women reported that most symptoms were not a problem (score, 0), certain musculoskeletal symptoms, vasomotor symptoms, and neuropsychological symptoms were at higher severities before AET initiation. Examples included joint pain (mean [SD] 2.7 [3.1]), muscle aches (mean [SD] 2.4 [3.0]), trouble sleeping (mean [SD] 2.2 [2.9]), and fatigue (mean [SD] 2.8 [2.7]). Patients also expressed mild severity of anxiety (mean [SD] 1.6 [2.4]) and being worried (mean [SD] 1.3 [2.2]), but fairly low severity of despair symptoms. During the first year of AET, patients experienced significant increases in most of the physical and psychological symptoms. Most notable were gastrointestinal symptoms (eg, weight change and bowel movement trouble), neuropsychological symptoms (eg, fatigue), vasomotor symptoms (eg, sweating and hot flashes), and musculoskeletal symptoms (eg, joint pain and muscle aches), with mean severity increasing by more than 1 point during the first year of AET.

**Figure 2.**
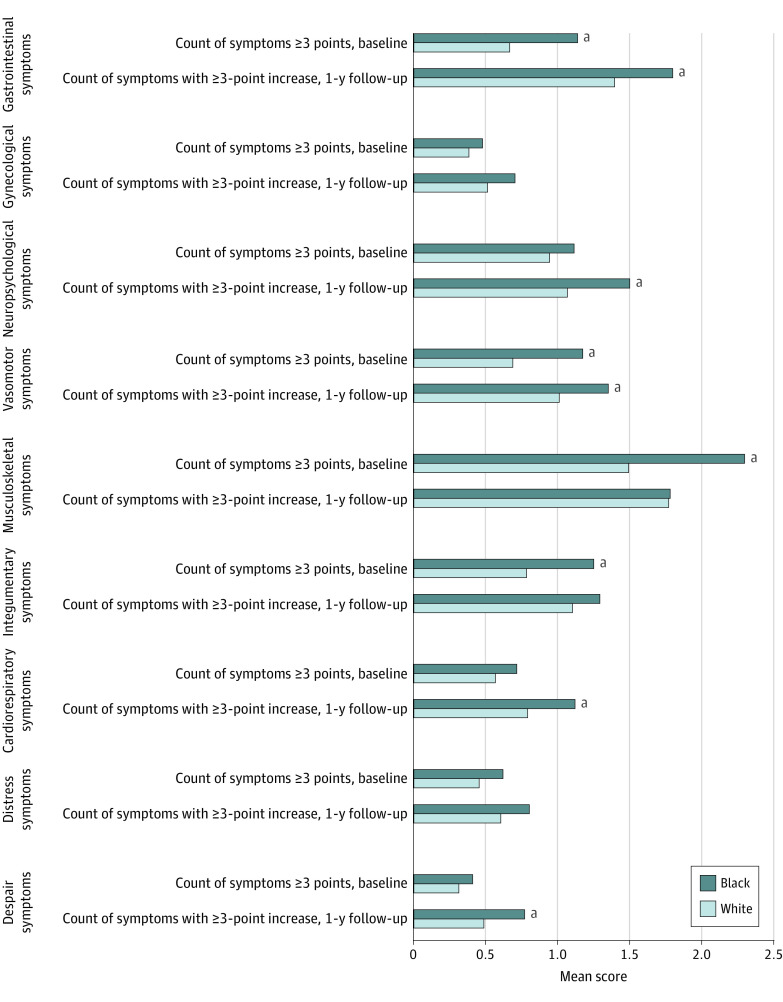
Mean Count of Symptoms at Baseline and During 1-Year Follow-up by Race Individual symptom items are scored on a 0 to 10 scale, where 0 indicates not a problem and 10 indicates as bad as possible. Baseline means were reported within 2 months before starting adjuvant endocrine therapy and changes from the baseline were determined by the highest severity symptoms reported during the 1-year follow-up period. ^a^Denotes comparison between Black and White (*P* < .05).

At baseline, Black patients reported more moderate severity symptoms for gastrointestinal, vasomotor, musculoskeletal, and integumentary clusters than White patients. During the first year of AET, Black women had more symptoms with a 3-point or greater increase in the following clusters: gastrointestinal (1.8 vs 1.4 items; *t*_275.93_ = 2.27; *P* = .02), neuropsychological (1.5 vs 1.1 items; *t*_267.33_ = 3.00; *P* = .003), cardiorespiratory (1.1 vs 0.8 items; *t*_276.98_ = 2.71; *P* = .007), and despair (0.8 vs 0.5 item; *t*_237.37_ = 2.03; *P* = .04) clusters (Figure 2).

### Symptom Burden and Adherence

The mean (SD) PDC by AET in the first year was 78.8% (27.2%) for Black and 82.3% (26.9%) for White women. After adjusting for sociodemographic and clinical characteristics, but not symptoms (Table 2), the PDC of Black women was 4.1 percentage points (95% CI, –1.9 to 10.1 percentage points) higher than White women. Each additional year of age was associated with a 0.5 percentage point higher adherence rate (95% CI, 0.2 to 0.8 percentage point) (eTable 4 in the Supplement). Models additionally adjusting for symptoms suggested that both baseline symptom levels and changes during AET were associated with lower adherence. (Table 2) For example, each additional baseline count of vasomotor symptoms decreased the adherence rate by 2.0 percentage points (95% CI, –3.8 to –0.3 percentage points), and each additional count of vasomotor symptoms with a 3-point or greater increase decreased the adherence rate by 2.0 percentage points (95% CI, –3.9 to –0.1 percentage points). Counts of intensified psychological symptoms during AET, including both distress and despair symptoms, were also significantly associated with lower adherence (distress, –2.6 percentage points [95% CI, –4.7 to –0.6 percentage points]; despair, –2.0 percentage points [95% CI, –3.8 to –0.2 percentage points]). After controlling for psychosocial symptoms, Black women showed a significantly higher adherence rate than White women (6.5 percentage points when adjusting for distress [95% CI, 0.1 to 12.8 percentage points]; 6.8 percentage points when adjusting for despair [95% CI, 0.5 to 13.2 percentage points]). Results for other patient characteristics associated with adherence are provided in eTable 4 and 5 in the Supplement.

**Table 2.** Adjusted Differences in AET Adherence (Proportion of Days Covered) Associated With Symptom Burden and Race

Model	Coefficient (95% CI)		
	Count of symptoms with ≥3-point increase, during AET	Count of moderate symptoms, baseline	Black (vs White)
Baseline model only^a^	NA	NA	4.1 (–1.9 to 10.1)
Baseline model adjusted for symptom cluster			
Gastrointestinal symptoms^b^	–1.1 (–2.4 to 0.1)	–1.6 (–3.2 to 0.0)	4.0 (–2.0 to 9.9)
Gynecological symptoms^b^	0.3 (–2.0 to 2.7)	–0.9 (–3.8 to 1.9)	3.9 (–2.1 to 9.9)
Neuropsychological symptoms^b^	–1.4 (–2.9 to 0.2)	–1.8 (–3.4 to –0.2)	3.9 (–2.0 to 9.9)
Vasomotor symptoms^b^	–2.0 (–3.8 to –0.3)	–2.0 (–3.9 to –0.1)	4.2 (–1.7 to 10.1)
Musculoskeletal symptoms^b^	–1.2 (–2.4 to –0.1)	–1.3 (–2.3 to –0.2)	3.7 (–2.2 to 9.7)
Integumentary symptoms^b^	–1.5 (–3.0 to 0.01)	–0.6 (–2.3 to 1.1)	4.2 (–1.7 to 10.2)
Cardiorespiratory symptoms^b^	–2.5 (–4.4 to –0.6)	–0.3 (–2.5 to 1.9)	3.8 (–2.1 to 9.8)
Distress symptoms^b^	–2.6 (–4.7 to –0.6)	–1.4 (–3.5 to 0.7)	6.5 (0.1 to 12.8)
Despair symptoms^b^	–2.0 (–3.8 to –0.2)	–0.6 (–2.7 to 1.5)	6.8 (0.5 to 13.2)

Abbreviations: AET, adjuvant endocrine therapy; NA, not applicable.

^a^ Baseline model was also adjusted for sociodemographic characteristics (age at diagnosis, race, neighborhood-level education, neighborhood-level income, residence state) and clinical characteristics (cancer stage, history of chemotherapy). Full models are provided in eTable 4 and eTable 5 in the Supplement.

^b^ Models also were adjusted for sociodemographic characteristics and clinical characteristics in baseline model, as well as corresponding symptom cluster (eg, neuropsychological symptoms, vasomotor symptoms, musculoskeletal symptoms, cardiorespiratory symptoms, distress symptoms). Full models are provided in eTable 4 and eTable 5 in the Supplement.

### Adherence Differences Explained by Differences in Symptom Burden (Kitagawa-Blinder-Oaxaca Decomposition)

Table 3 shows results from the Kitagawa-Blinder-Oaxaca decomposition of net difference in observed adherence rates by race, which was 3.5 percentage points higher for White than Black women (95% CI, –1.6 to 8.6 percentage points). Explained differences (Table 3) were consistently positive and larger than the net difference, ranging from 4.9 percentage points to 8.1 percentage points. When psychological symptoms were included in the model, the explained differences were the largest (distress, 7.5 percentage points [95% CI, 1.2 to 13.9 percentage points]; despair, 8.1 percentage points [95% CI, 1.8 to 14.4 percentage points]). The remaining unexplained differences were negative, ranging from −1.5 to −5.5 percentage points.

**Table 3.** Kitagawa-Blinder-Oaxaca Decomposition of AET Adherence (Proportion of Days Covered)

Model	Black	White	Mean (95% CI)		
			Net difference (White) - (Black)	Explained Difference	Unexplained Difference
Baseline model^a^	78.8	82.3	3.5 (–1.6 to 8.6)	5.7 (–0.1 to 11.6)	–2.3 (–9.9 to 5.4)
Baseline model adjusted for symptom clusters					
Gastrointestinal symptoms^b^	78.8	82.3	3.5 (–1.6 to 8.6)	5.1 (–0.1 to 10.8)	–1.6 (–9.1 to 6.0)
Gynecological symptoms^b^	78.8	82.5	3.6 (–1.5 to 8.7)	5.7 (–0.1 to 11.5)	–2.1 (–9.7 to 5.6)
Neuropsychological symptoms^b^	78.8	82.3	3.5 (–1.6 to 8.6)	5.8 (–0.1 to 11.8)	–2.4 (–10.1 to 5.4)
Vasomotor symptoms^b^	78.8	82.3	3.5 (–1.6 to 8.6)	5.6 (–0.2 to 11.4)	–2.1 (–9.8 to 5.5)
Musculoskeletal symptoms^b^	78.8	82.3	3.5 (–1.6 to 8.6)	4.9 (–0.9 to 10.8)	–1.5 (–9.1 to 6.2)
Integumentary symptoms^b^	78.8	82.3	3.5 (–1.6 to 8.6)	5.6 (–0.2 to 11.3)	–2.1 (–9.7 to 5.5)
Cardiorespiratory symptoms^b^	78.8	82.3	3.5 (–1.6 to 8.6)	5.4 (–0.4 to 11.2)	–1.9 (–9.5 to 5.7)
Distress symptoms^b^	79.1	81.7	2.6 (–2.9 to 8.1)	7.5 (1.2 to 13.9)	–4.9 (–13.0 to 3.2)
Despair symptoms^b^	79.1	81.7	2.6 (–2.9 to 8.0)	8.1 (1.8 to 14.4)	–5.5 (–13.6 to 2.6)

Abbreviation: AET, adjuvant endocrine therapy.

^a^ Baseline model was adjusted for sociodemographic characteristics (age at diagnosis, race, neighborhood-level education, neighborhood-level income, residence state, insurance status), clinical characteristics (cancer stage, history of chemotherapy to AET initiation), and first AET drug type.

^b^ Model was adjusted for sociodemographic characteristics and clinical characteristics in baseline model, as well as corresponding symptom cluster (eg, neuropsychological symptoms, vasomotor symptoms, musculoskeletal symptoms, cardiorespiratory symptoms, distress symptoms).

## Discussion

To our knowledge, this cohort study is the first using longitudinal patient-reported data to compare physical and psychological symptom burden during AET among Black and White women with early-stage breast cancer and to examine differences in the association between symptom burden and AET adherence by race. Black patients reported significantly higher symptom burden at baseline and during AET. Although Black women showed slightly lower adherence than White women, if they shared similar sociodemographic and clinical characteristics, as well as symptom burden, Black women would have higher adherence than White women.

We found that patients experienced significant increases in most symptoms, especially gastrointestinal symptoms, neuropsychological symptoms, vasomotor symptoms, and musculoskeletal symptoms after starting AET. These symptoms were more commonly prevalent at baseline and more likely to increase during AET among Black women. Moreover, a higher proportion of Black women experience worsening despair symptoms than White women. Despite routine symptom collection at WCCRI, a higher symptom burden among Black women suggests disproportionate undertreatment of symptoms.^17^ Our results suggest that systematic assessment of symptoms is insufficient to produce equitable outcomes within the health care system. Women in settings where symptoms are not systematically assessed may experience even larger disparities in symptom burden and cancer outcomes under the powerful influence of structural racism.^48,49,50,51^

Previous research^5,6^ shows that White women have higher adherence to AETs compared with women of other races. Similarly, we found lower adherence rates among Black women (78.8%) than White women (82.3%) despite fairly high overall adherence in the first year of AET use. Consistent with previous literature, younger age was associated with lower adherence.^3,52^ Both baseline symptoms and changes during AET were associated with significantly lower adherence. Similarly, prior research found that patients who had already accumulated a high symptom burden from previous treatment were more likely to discontinue AET.^52^ AET itself was associated with additional toxicity, further impacting patients’ adherence. Each additional count of moderate severity symptoms at baseline or each additional count of symptoms with 3-point or greater increase is associated with a decrease in AET coverage by 4 to 9 days. This points to the importance of continuous symptom monitoring and patient communication throughout the treatment period.

Our Kitagawa-Blinder-Oaxaca decomposition found a positive explained difference by race that was consistently larger than the net observed difference by race. This consistent trend means that differences in Black and White women’s baseline characteristics (eg, socioeconomic status, age, and cancer stage) and symptom severity explained the observed differences in adherence rate and estimated an even larger gap in adherence rate between the 2 groups. The remaining unexplained differences were negative, meaning that if Black and White women shared the same baseline characteristics and symptom burden, White women would have lower adherence rates than Black women. This is contrary to the perception that Black patients are more likely to be nonadherent to treatment.^53^ Instead, it suggests that Black women may have some protective characteristics that make them more resilient to adverse symptoms and the harmful effects of systemic racism.^51,54^

Addressing differences in psychological symptoms between Black and White women may be as important as addressing differences in physical symptoms to reduce disparities in treatment adherence. Importantly, in addition to sociodemographic and clinical characteristics, differences in physical and psychological symptoms may be due to institutional bias. The inclusion of these symptoms in the decomposition model sheds light on how addressing institutional bias could help eliminate disparities in AET adherence. Our results offer hope that we may be able to mitigate disparities related to treatment adherence and subsequent breast cancer outcomes with continuous racial bias training, infrastructure investment, and more equitable symptom monitoring and management.

### Limitations

Our study has limitations. First, this is a cohort study with observational data and, therefore, we cannot establish causal relationships between symptoms and adherence. Second, we measured adherence using a claim-based PDC measure and restricted it to the first year of AET. Even though PDC may overestimate actual adherence, previous validation studies^33,35^ showed that it was usually consistent with the rate at which patients consume drugs and predictive of health outcomes. Future research should extend evaluation to longer follow-up periods and assess alternative adherence measures. Furthermore, our study is restricted to women with a diagnosis of hormone receptor-positive, early-stage breast cancer from a single cancer center in 2007 to 2015 and continuously covered by Medicare and/or Medicaid. We compared patient characteristics with the larger cohort without requiring continuous insurance coverage or having PCM reports, and observed that the larger cohort was similar to our final sample (eTable 6 in the Supplement). Still, the inclusion criteria may induce selection bias, and our included patients might be generally older and have lower income than the larger population with breast cancer who initiated AET.s Our finding may not generalize to other disease conditions, study settings, patients with private insurance types, or patients with more recent diagnoses. Continuing efforts are needed to understand the racial differences in treatment-related symptom burdens and AET adherence with more recent data and longer follow-ups.

## Conclusions

We found that Black women with early-stage hormone receptor-positive breat cancer reported more severe symptoms at baseline and more frequent increases in symptom burden during the first year of AET treatment. Decomposition results show that if Black women shared similar characteristics and symptom burden as White women, they would have higher adherence than White women. Better symptom management and more focus on the management of psychological symptoms among Black women could improve AET adherence and reduce racial disparities in cancer outcomes.

## Notes

Supplement. **eTable 1. **List of Adjuvant Endocrine Therapy Drugs **eFigure. **Sample Selection Flow Chart **eTable 2. **Illustration of Individual Scale **eTable 3. **List of Individual Physical and Psychological Symptoms **eTable 4. **Multivariable Linear Regression for AET Adherence Adjusted for Physical Symptoms Clusters **eTable 5. **Multivariable Linear Regression for AET Adherence Adjusted for Psychological Symptoms Clusters **eTable 6. **Sample Characteristics of the Larger Cohort Without Requiring Continuous Insurance Enrollment or Having PCM Reports

## References

[1] Tjan-Heijnen VCG, van Hellemond IEG, Peer PGM, ; Dutch Breast Cancer Research Group (BOOG) for the DATA Investigators. Extended adjuvant aromatase inhibition after sequential endocrine therapy (DATA): a randomised, phase 3 trial. Lancet Oncol. 2017;18(11):1502-1511. doi:10.1016/S1470-2045(17)30600-9 29031778

[2] Goss PE, Ingle JN, Pritchard KI, . Extending aromatase-inhibitor adjuvant therapy to 10 years. N Engl J Med. 2016;375(3):209-219. doi:10.1056/NEJMoa1604700 27264120 PMC5024713

[3] Yussof I, Mohd Tahir NA, Hatah E, Mohamed Shah N. Factors influencing five-year adherence to adjuvant endocrine therapy in breast cancer patients: a systematic review. The Breast. 2022;62:22-35. doi:10.1016/j.breast.2022.01.012 35121501 PMC8818734

[4] Chlebowski RT, Kim J, Haque R. Adherence to endocrine therapy in breast cancer adjuvant and prevention settings. Cancer Prev Res (Phila). 2014;7(4):378-387. doi:10.1158/1940-6207.CAPR-13-0389 24441675 PMC11649036

[5] Heiney SP, Truman S, Babatunde OA, . Racial and geographic disparities in endocrine therapy adherence among younger breast cancer survivors. Am J Clin Oncol. 2020;43(7):504-509. doi:10.1097/COC.0000000000000696 32251120 PMC7316591

[6] Farias AJ, Wu W-H, Du XL. Racial differences in long-term adjuvant endocrine therapy adherence and mortality among Medicaid-insured breast cancer patients in Texas: findings from TCR-Medicaid linked data. BMC Cancer. 2018;18(1):1214. doi:10.1186/s12885-018-5121-z 30514270 PMC6280479

[7] Wheeler SB, Spencer J, Pinheiro LC, . Endocrine therapy nonadherence and discontinuation in black and white women. J Natl Cancer Inst. 2019;111(5):498-508. doi:10.1093/jnci/djy136 30239824 PMC6510227

[8] Kimmick G, Edmond SN, Bosworth HB, . Medication taking behaviors among breast cancer patients on adjuvant endocrine therapy. Breast. 2015;24(5):630-636. doi:10.1016/j.breast.2015.06.010 26189978 PMC4824055

[9] Wagner LI. Patient-reported outcomes bridge an important gap in identifying risk for early endocrine therapy discontinuation. J Natl Cancer Inst. 2021;113(8):945-947. doi:10.1093/jnci/djab024 33585932

[10] Toivonen KI, Williamson TM, Carlson LE, Walker LM, Campbell TS. Potentially modifiable factors associated with adherence to adjuvant endocrine therapy among breast cancer survivors: a systematic review. Cancers (Basel). 2020;13(1):107. doi:10.3390/cancers13010107 33561076 PMC7794693

[11] Check DK, Chawla N, Kwan ML, . Understanding racial/ethnic differences in breast cancer-related physical well-being: the role of patient-provider interactions. Breast Cancer Res Treat. 2018;170(3):593-603. doi:10.1007/s10549-018-4776-0 29623576 PMC6528788

[12] McFarland DC, Shaffer KM, Tiersten A, Holland J. Physical symptom burden and its association with distress, anxiety, and depression in breast cancer. Psychosomatics. 2018;59(5):464-471. doi:10.1016/j.psym.2018.01.005 29525522 PMC6067989

[13] Samuel CA, Schaal J, Robertson L, . Racial differences in symptom management experiences during breast cancer treatment. Support Care Cancer. 2018;26(5):1425-1435. doi:10.1007/s00520-017-3965-4 29150730 PMC6363352

[14] Tejeda S, Stolley MR, Vijayasiri G, . Negative psychological consequences of breast cancer among recently diagnosed ethnically diverse women. Psychooncology. 2017;26(12):2245-2252. doi:10.1002/pon.4456 28499328

[15] Yee MK, Sereika SM, Bender CM, Brufsky AM, Connolly MC, Rosenzweig MQ. Symptom incidence, distress, cancer-related distress, and adherence to chemotherapy among African American women with breast cancer. Cancer. 2017;123(11):2061-2069. doi:10.1002/cncr.30575 28199006

[16] Hu X, Chehal PK, Kaplan C, . Characterization of clinical symptoms by race among women with early-stage, hormone receptor-positive breast cancer before starting chemotherapy. JAMA Netw Open. 2021;4(6):e2112076. doi:10.1001/jamanetworkopen.2021.12076 34061200 PMC8170541

[17] Hoffman KM, Trawalter S, Axt JR, Oliver MN. Racial bias in pain assessment and treatment recommendations, and false beliefs about biological differences between blacks and whites. Proc Natl Acad Sci U S A. 2016;113(16):4296-4301. doi:10.1073/pnas.1516047113 27044069 PMC4843483

[18] Pletcher MJ, Kertesz SG, Kohn MA, Gonzales R. Trends in opioid prescribing by race/ethnicity for patients seeking care in US emergency departments. JAMA. 2008;299(1):70-78. doi:10.1001/jama.2007.64 18167408

[19] Nong P, Raj M, Creary M, Kardia SLR, Platt JE. Patient-reported experiences of discrimination in the US health care system. JAMA Netw Open. 2020;3(12):e2029650-e2029650. doi:10.1001/jamanetworkopen.2020.29650 33320264 PMC7739133

[20] Milner A, Jumbe S. Using the right words to address racial disparities in COVID-19. Lancet Public Health. 2020;5(8):e419-e420. doi:10.1016/S2468-2667(20)30162-6 32707127 PMC7373398

[21] Goel N, Westrick AC, Bailey ZD, . Structural racism and breast cancer-specific survival: impact of economic and racial residential segregation. Ann Surg. 2022;275(4):776-783. doi:10.1097/SLA.0000000000005375 35081560 PMC9102835

[22] Vela MB, Erondu AI, Smith NA, Peek ME, Woodruff JN, Chin MH. Eliminating explicit and implicit biases in health care: evidence and research needs. Annu Rev Public Health. 2022;43:477-501. doi:10.1146/annurev-publhealth-052620-103528 35020445 PMC9172268

[23] Machery E, Faucher L. Social construction and the concept of race. Philos Sci. 2005;72(5):1208-1219. doi:10.1086/508966

[24] Sadigh G, Gray RJ, Sparano JA, . Assessment of racial disparity in survival outcomes for early hormone receptor-positive breast cancer after adjusting for insurance status and neighborhood deprivation: a post hoc analysis of a randomized clinical trial. JAMA Oncol. 2022;8(4):579-586. doi:10.1001/jamaoncol.2021.7656 35175284 PMC8855314

[25] Tucker-Seeley RD. Social determinants of health and disparities in cancer care for Black people in the United States. JCO Oncol Pract. 2021;17(5):261-263. doi:10.1200/OP.21.00229 33974819

[26] Giesinger JM, Wintner LM, Zabernigg A, . Assessing quality of life on the day of chemotherapy administration underestimates patients’ true symptom burden. BMC Cancer. 2014;14(1):758. doi:10.1186/1471-2407-14-758 25305067 PMC4198707

[27] Pinheiro LC, Samuel CA, Reeder-Hayes KE, Wheeler SB, Olshan AF, Reeve BB. Understanding racial differences in health-related quality of life in a population-based cohort of breast cancer survivors. Breast Cancer Res Treat. 2016;159(3):535-543. doi:10.1007/s10549-016-3965-y 27585477 PMC5031495

[28] Bulls HW, Chang P-H, Brownstein NC, . Patient-reported symptom burden in routine oncology care: examining racial and ethnic disparities. Cancer Rep. 2022;5(3):e1478. doi:10.1002/cnr2.1478 PMC8955049 34165256

[29] Sheppard VB, Sutton AL, Hurtado-de-Mendoza A, . Race and patient-reported symptoms in adherence to adjuvant endocrine therapy: a report from the women’s hormonal initiation and persistence study. Cancer Epidemiol Biomarkers Prev. 2021;30(4):699-709. doi:10.1158/1055-9965.EPI-20-0604 33514603 PMC8330157

[30] Fortner B, Baldwin S, Schwartzberg L, Houts AC. Validation of the Cancer Care Monitor items for physical symptoms and treatment side effects using expert oncology nurse evaluation. J Pain Symptom Manage. 2006;31(3):207-214. doi:10.1016/j.jpainsymman.2005.07.009 16563315

[31] Fortner B, Okon T, Schwartzberg L, Tauer K, Houts AC. The Cancer Care Monitor: psychometric content evaluation and pilot testing of a computer administered system for symptom screening and quality of life in adult cancer patients. J Pain Symptom Manage. 2003;26(6):1077-1092. doi:10.1016/j.jpainsymman.2003.04.003 14654260

[32] Ringash J, O’Sullivan B, Bezjak A, Redelmeier DA. Interpreting clinically significant changes in patient-reported outcomes. Cancer. 2007;110(1):196-202. doi:10.1002/cncr.22799 17546575

[33] Karve S, Cleves MA, Helm M, Hudson TJ, West DS, Martin BC. Prospective validation of eight different adherence measures for use with administrative claims data among patients with schizophrenia. Value Health. 2009;12(6):989-995. doi:10.1111/j.1524-4733.2009.00543.x 19402852

[34] Yeaw J, Benner JS, Walt JG, Sian S, Smith DB. Comparing adherence and persistence across 6 chronic medication classes. J Manag Care Pharm. 2009;15(9):728-740. doi:10.18553/jmcp.2009.15.9.728 19954264 PMC10441195

[35] Grymonpre R, Cheang M, Fraser M, Metge C, Sitar DS. Validity of a prescription claims database to estimate medication adherence in older persons. Med Care. 2006;44(5):471-477. doi:10.1097/01.mlr.0000207817.32496.cb 16641666

[36] Andersen RM. Revisiting the behavioral model and access to medical care: does it matter? J Health Soc Behav. 1995;36(1):1-10. doi:10.2307/2137284 7738325

[37] Cleeland CS, Zhao F, Chang VT, . The symptom burden of cancer: evidence for a core set of cancer-related and treatment-related symptoms from the Eastern Cooperative Oncology Group Symptom Outcomes and Practice Patterns study. Cancer. 2013;119(24):4333-4340. doi:10.1002/cncr.28376 24114037 PMC3860266

[38] Hamer J, McDonald R, Zhang L, . Quality of life (QOL) and symptom burden (SB) in patients with breast cancer. Supportive Care Cancer. 2017;25(2):409-419. doi:10.1007/s00520-016-3417-6 27696078

[39] Nurgalieva ZZ, Franzini L, Morgan RO, Vernon SW, Liu CC, Du XL. Impact of timing of adjuvant chemotherapy initiation and completion after surgery on racial disparities in survival among women with breast cancer. Med Oncol. 2013;30(1):419. doi:10.1007/s12032-012-0419-1 23292872

[40] Green AK, Aviki EM, Matsoukas K, Patil S, Korenstein D, Blinder V. Racial disparities in chemotherapy administration for early-stage breast cancer: a systematic review and meta-analysis. Breast Cancer Res Treat. 2018;172(2):247-263. doi:10.1007/s10549-018-4909-5 30094552 PMC6958704

[41] Fedewa SA, Ward EM, Stewart AK, Edge SB. Delays in adjuvant chemotherapy treatment among patients with breast cancer are more likely in African American and Hispanic populations: a national cohort study 2004-2006. J Clin Oncol. 2010;28(27):4135-4141. doi:10.1200/JCO.2009.27.2427 20697082

[42] Hurtado-de-Mendoza A, Cabling ML, Lobo T, Dash C, Sheppard VB. Behavioral interventions to enhance adherence to hormone therapy in breast cancer survivors: a systematic literature review. Clin Breast Cancer. 2016;16(4):247-255.e3. doi:10.1016/j.clbc.2016.03.006 27133733 PMC4969158

[43] Garreau JR, Delamelena T, Walts D, Karamlou K, Johnson N. Side effects of aromatase inhibitors versus tamoxifen: the patients’ perspective. Am J Surg. 2006;192(4):496-498. doi:10.1016/j.amjsurg.2006.06.018 16978958

[44] Oaxaca R. Male-female wage differentials in urban labor markets. Int Econ Rev. 1973;14(3):693-709. doi:10.2307/2525981

[45] Blinder AS. Wage discrimination: reduced form and structural estimates. J Hum Resour. 1973;8(4):436-455. doi:10.2307/144855

[46] Jann B. The Blinder-Oaxaca decomposition for linear regression models. Stata J. 2008;8(4):453-479. doi:10.1177/1536867X0800800401

[47] Kitagawa EM. Components of a difference between two rates. J Am Stat Assoc. 1955;50(272):1168-1194. doi:10.2307/2281213

[48] Smedley A, Smedley BD. Race as biology is fiction, racism as a social problem is real: anthropological and historical perspectives on the social construction of race. Am Psychol. 2005;60(1):16-26. doi:10.1037/0003-066X.60.1.16 15641918

[49] Williams DR, Mohammed SA, Shields AE. Understanding and effectively addressing breast cancer in African American women: unpacking the social context. Cancer. 2016;122(14):2138-2149. doi:10.1002/cncr.29935 26930024 PMC5588632

[50] Beyer KMM, Zhou Y, Laud PW, . Mortgage lending bias and breast cancer survival among older women in the United States. J Clin Oncol. 2021;39(25):2749-2757. doi:10.1200/JCO.21.00112 34129388 PMC8407650

[51] White-Means S, Rice M, Dapremont J, Davis B, Martin J. African American women: surviving breast cancer mortality against the highest odds. Int J Environ Res Public Health. 2015;13(1):ijerph13010006. doi:10.3390/ijerph13010006 26703655 PMC4730397

[52] Hershman DL, Neugut AI, Moseley A, . Patient-reported outcomes and long-term nonadherence to aromatase inhibitors. J Natl Cancer Inst. 2021;113(8):989-996. doi:10.1093/jnci/djab022 33629114 PMC8328987

[53] van Ryn M, Burke J. The effect of patient race and socio-economic status on physicians' perceptions of patients. Soc Sci Med. 2000;50(6):813-828. doi:10.1016/S0277-9536(99)00338-X 10695979

[54] Culver JL, Arena PL, Antoni MH, Carver CS. Coping and distress among women under treatment for early stage breast cancer: comparing African Americans, Hispanics and non-Hispanic Whites. Psychooncology. 2002;11(6):495-504. doi:10.1002/pon.615 12476431

