# Supplementary material for: Racial Differences in Patient-Reported Symptoms and Adherence to Adjuvant Endocrine Therapy Among Women With Early-Stage, Hormone Receptor–Positive Breast Cancer
Source: JAMA Netw Open. 2022 Aug 10;5(8):e2225485. doi: 10.1001/jamanetworkopen.2022.25485 (PMC9366541; doi:10.1001/jamanetworkopen.2022.25485)
Supplement: Supplement. — eTable 1. List of Adjuvant Endocrine Therapy Drugs eFigure. Sample Selection Flow Chart eTable 2. Illustration of Individual Scale eTable 3. List of Individual Physical and Psychological Symptoms eTable 4. Multivariable Linear Regression for AET Adherence Adjusted for Physical Symptoms Clusters eTable 5. Multivariable Linear Regression for AET Adherence Adjusted for Psychological Symptoms Clusters eTable 6. Sample Characteristics of the Larger Cohort Without Requiring Continuous Insurance Enrollment or Having PCM Reports [file jamanetwopen-e2225485-s001.pdf]

Supplemental Online Content

Hu X, Walker MS, Stepanski E, et al. Race differences in patient-reported symptoms and adherence to adjuvant endocrine therapy among women with early-stage, hormone receptor–positive breast cancer. *JAMA Netw Open*. 2022;5(8):e2225485.
doi:10.1001/jamanetworkopen.2022.25485

eTable 1. List of Adjuvant Endocrine Therapy Drugs

eFigure. Sample Selection Flow Chart

eTable 2. Illustration of Individual Scale

eTable 3. List of Individual Physical and Psychological Symptoms

eTable 4. Multivariable Linear Regression for AET Adherence Adjusted for Physical Symptoms Clusters

eTable 5. Multivariable Linear Regression for AET Adherence Adjusted for Psychological Symptoms Clusters

eTable 6. Sample Characteristics of the Larger Cohort Without Requiring Continuous Insurance Enrollment or Having PCM Reports

This supplemental material has been provided by the authors to give readers additional information about their work.

© 2022 Hu X et al. *JAMA Network Open.*

eTable 1. List of Adjuvant Endocrine Therapy Drugs

**Generic Name**	**Brand Name**
Anastrozole	Arimidex
Exemestane	Aromasin
Letrozole	Femara
Tamoxifen	Soltamox
Tamoxifen	Nolvadex

© 2022 Hu X et al. *JAMA Network Open.*

eFigure. Sample Selection Flow Chart

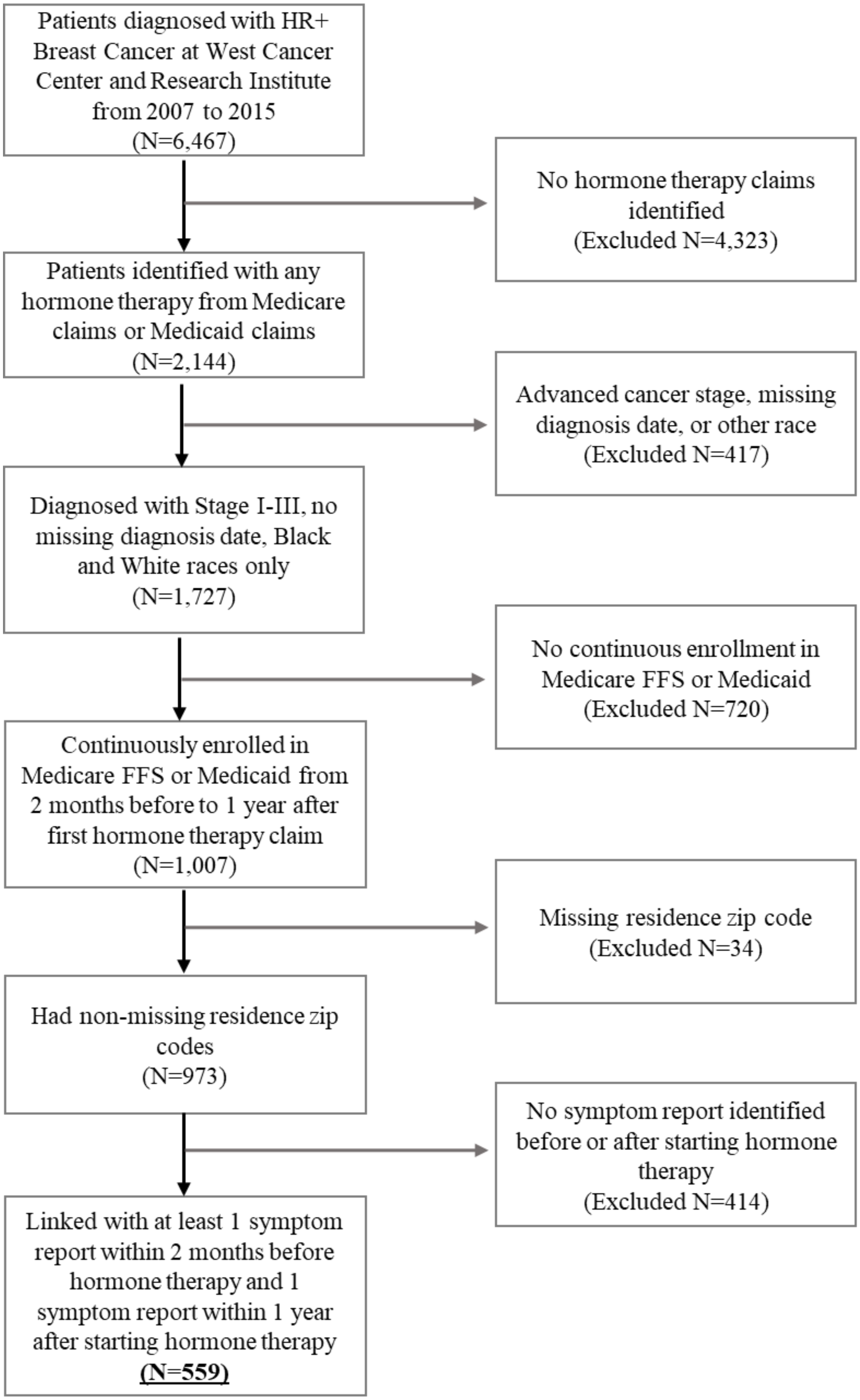

© 2022 Hu X et al. *JAMA Network Open.*

eTable 2. Illustration of Individual Scale

INSTRUCTIONS: Touch red dot on the line that best describes how bad the symptom has been for you during the past week including today.

Not a problem	Mild problem	Moderate problem	Severe problem	As bad as possible
**0**	**1 2 3**	**4 5 6**	**7 8 9**	**10**
**O**	**O O O**	**O O O**	**O O O**	**O**

Fatigue, tiredness, or weakness

Rash or dry skin or itching

Trouble sleeping

Daytime sleepiness

Physical pain

Sweating

Trouble breathing or coughing

Trouble focusing or concentrating

Problems with urination

Numbness or tingling

Reduced sexual enjoyment, interest, or performance

© 2022 Hu X et al. *JAMA Network Open.*

eTable 3. List of Individual Physical and Psychological Symptoms

Symptom Cluster	Individual Item
Gastrointestinal symptoms	Change in Weight
	Nausea or vomiting
	Trouble with bowel movements
	Change in appetite
	Dry mouth
	Change in the taste of food
	Sore throat or trouble swallowing
	Mouth sores
Gynecologic symptoms	Vaginal itching
	Vaginal bleeding/discharge
	Vaginal dryness
	Reduced sexual enjoyment, interest, or performance
	Breast tenderness/discharge
	New lump/mass
	Menstrual pain/cramping
Neuropsychologic symptoms	Headache
	Dizziness
	Fatigue, tiredness, or weakness
	Trouble focusing or concentrating
	Difficulty hearing
	Memory loss
Vasomotor symptoms	Sweating
	Hot flashes/flushes
	Trouble sleeping
	Daytime sleepiness
	Fever or Chills
Musculoskeletal symptoms	Joint pain
	Muscle aches
	Physical Pain
	Numbness or tingling
	Problems with urination
	Trouble seeing
	Burning sensation in hands/feet
	Weakness of body parts
Integumentary symptoms	Rash or dry skin or itching
	Hair loss
	Bruising
	Easy bleeding
	Swelling
	Dry eyes
	Eyes tearing
	Hives
	Nail changes
Cardiorespiratory symptoms	Trouble breathing or coughing
	Sinus Problems
	Difficulty breathing
	Chest pain
	Rapid heart beat

© 2022 Hu X et al. *JAMA Network Open.*

	Heartburn
Distress Symptoms	I feel nervous, tense, or anxious
	I am worried
	I cry a lot or feel like crying
	I am sad or depressed
Despair Symptoms	I feel worthless
	I have lost interest in things
	I feel guilty
	I think I would be better of dead
	I feel helpless
	I have lost interest in people
	I feel hopeless

© 2022 Hu X et al. *JAMA Network Open.*

eTable 4. Multivariable Linear Regression for AET Adherence Adjusted for Physical Symptoms Clusters

	Baseline Model[a] (1)	Baseline Model +Gastrointestinal Symptoms[b] (2)	Baseline Model + Gynecologic Symptoms[b] (3)	Baseline Model +Neuropsychologic Symptoms[b] (4)	Baseline Model + Vasomotor Symptoms[b] (5)	Baseline Model +Musculoskeletal Symptoms[b] (6)	Baseline Model +Integumentary Symptoms[b] (7)	Baseline Model +Cardiorespiratory Symptoms[b] (8)
	Coefficient (95% CI)	Coefficient (95% CI)	Coefficient (95% CI)	Coefficient (95% CI)	Coefficient (95% CI)	Coefficient (95% CI)	Coefficient (95% CI)	Coefficient (95% CI)
**Count of symptoms with ≥ 3-point increase, during AET**		-1.1 (-2.4, 0.1)	0.3 (-2.0, 2.7)	-1.4 (-2.9, 0.2)	-2.0 (-3.8, -0.3)	-1.2 (-2.4, -0.1)	-1.5 (-3.0, 0.01)	-2.5 (-4.4, -0.6)
**Count of moderate symptoms, baseline**		-1.6 (-3.2, 0.0)	-0.9 (-3.8, 1.9)	-1.8 (-3.4, -0.2)	-2.0 (-3.9, -0.1)	-1.3 (-2.3, -0.2)	-0.6 (-2.3, 1.1)	-0.3 (-2.5, 1.9)
**Race**								
White	Ref	Ref	Ref	Ref	Ref	Ref	Ref	Ref
Black	4.1 (-1.9, 10.1)	4.0 (-2.0, 9.9)	3.9 (-2.1, 9.9)	3.9 (-2.0, 9.9)	4.2 (-1.7, 10.1)	3.7 (-2.2, 9.7)	4.2 (-1.7, 10.2)	3.8 (-2.1, 9.8)
**Age at Diagnosis**	0.5 (0.2, 0.8)	0.4 (0.2, 0.7)	0.5 (0.2, 0.8)	0.5 (0.2, 0.7)	0.4 (0.2, 0.7)	0.5 (0.2, 0.7)	0.5 (0.2, 0.8)	0.5 (0.2, 0.7)
**Cancer Stage**								
Stage I	Ref	Ref	Ref	Ref	Ref	Ref	Ref	Ref
Stage II	3.1 (-1.9, 8.2)	3.6 (-1.4, 8.6)	2.8 (-2.2, 7.9)	3.6 (-1.5, 8.6)	3.5 (-1.5, 8.5)	3.5 (-1.5, 8.6)	3.5 (-1.6, 8.5)	3.6 (-1.4, 8.7)
Stage III	2.0 (-6.0, 9.9)	2.5 (-5.4, 10.5)	1.7 (-6.3, 9.6)	2.2 (-5.7, 10.1)	2.0 (-5.9, 10.0)	2.4 (-5.5, 10.3)	2.4 (-5.6, 10.3)	2.9 (-5.0, 10.9)
**Neighborhood Level High School Education**	-0.1 (-0.6, 0.4)	-0.1 (-0.5, 0.4)	-0.1 (-0.6, 0.3)	-0.1 (-0.6, 0.4)	-0.1 (-0.6, 0.4)	-0.1 (-0.5, 0.4)	-0.1 (-0.6, 0.4)	-0.1 (-0.6, 0.4)
**Neighborhood Level Household Income**								
<$35,000	Ref	Ref	Ref	Ref	Ref	Ref	Ref	Ref
$35,000-$44,999	-2.4 (-9.4, 4.5)	-2.7 (-9.6, 4.2)	-2.6 (-9.6, 4.3)	-2.8 (-9.7, 4.1)	-2.4 (-9.3, 4.5)	-3.1 (-10.0, 3.8)	-2.1 (-9.0, 4.8)	-2.6 (-9.5, 4.3)
$45,000-$54,999	3.6 (-5.0, 12.3)	3.0 (-5.6, 11.6)	3.5 (-5.2, 12.1)	3.4 (-5.1, 12.0)	3.1 (-5.5, 11.7)	3.1 (-5.5, 11.6)	3.5 (-5.2, 12.1)	2.8 (-5.8, 11.4)
>=$55,000	3.3 (-7.5, 14.0)	2.1 (-8.6, 12.8)	3.2 (-7.5, 13.9)	2.5 (-8.1, 13.2)	3.0 (-7.7, 13.6)	1.6 (-9.1, 12.3)	2.7 (-8.0, 13.4)	2.3 (-8.4, 13.0)
**State of Residence**								
Tennessee	Ref	Ref	Ref	Ref	Ref	Ref	Ref	Ref
Arkansas	1.8 (-9.6, 13.3)	1.9 (-9.5, 13.3)	1.6 (-9.8, 13.1)	2.0 (-9.4, 13.4)	2.8 (-8.6, 14.2)	2.3 (-9.1, 13.7)	2.4 (-9.1, 13.9)	3.0 (-8.4, 14.5)
Mississippi	-2.9 (-8.5, 2.7)	-2.5 (-8.1, 3.1)	-2.6 (-8.2, 2.9)	-2.3 (-7.8, 3.3)	-2.2 (-7.8, 3.3)	-2.3 (-7.8, 3.3)	-2.1 (-7.7, 3.5)	-2.1 (-7.7, 3.5)
Other	4.6 (-10.7, 20.0)	4.4 (-10.9, 19.6)	4.5 (-10.8, 19.7)	4.5 (-10.7, 19.8)	4.0 (-11.2, 19.2)	3.0 (-12.3, 18.2)	4.5 (-10.8, 19.8)	5.1 (-10.2, 20.4)
**History of chemotherapy**								
No chemotherapy	Ref	Ref	Ref	Ref	Ref	Ref	Ref	Ref
Chemotherapy within 180 days before or after AET initiation	5.1 (-1.4, 11.6)	6.0 (-0.5, 12.5)	4.9 (-1.5, 11.4)	5.6 (-0.8, 12.0)	5.8 (-0.7, 12.2)	5.6 (-0.9, 12.0)	5.9 (-0.6, 12.5)	5.2 (-1.2, 11.7)
Chemotherapy beyond 180 days before AET	-2.7 (-9.1, 3.7)	-2.1 (-8.5, 4.3)	-2.7 (-9.0, 3.7)	-2.1 (-8.5, 4.2)	-2.1 (-8.5, 4.2)	-1.4 (-7.8, 5.0)	-2.2 (-8.7, 4.3)	-2.3 (-8.7, 4.0)
**Insurance type**								
Medicare Only	Ref	Ref	Ref	Ref	Ref	Ref	Ref	Ref
Medicaid or Dual	-6.0 (-13.7, 1.6)	-4.9 (-12.6, 2.7)	-6.2 (-13.8, 1.5)	-5.2 (-12.8, 2.5)	-4.3 (-12.0, 3.3)	-4.8 (-12.4, 2.9)	-5.3 (-13.0, 2.4)	-4.6 (-12.3, 3.1)
**First AET type**								
Tamoxifen	Ref	Ref	Ref	Ref	Ref	Ref	Ref	Ref
Anastrozole	4.4 (-1.8, 10.6)	4.5 (-1.7, 10.7)	3.6 (-2.6, 9.8)	4.3 (-1.8, 10.5)	4.8 (-1.4, 10.9)	4.6 (-1.5, 10.8)	4.6 (-1.6, 10.8)	4.6 (-1.5, 10.8)
Exemestane	-1.3 (-10.3, 7.7)	-1.7 (-10.7, 7.2)	-2.1 (-11.1, 6.9)	-1.9 (-10.8, 7.0)	-1.8 (-10.7, 7.1)	-1.7 (-10.6, 7.2)	-1.6 (-10.5, 7.4)	-1.5 (-10.4, 7.5)
Letrozole	-0.2 (-7.7, 7.2)	0.5 (-7.0, 7.9)	-0.9 (-8.4, 6.6)	0.5 (-6.9, 8.0)	0.8 (-6.6, 8.3)	0.9 (-6.6, 8.3)	0.5 (-7.0, 8.0)	0.7 (-6.8, 8.2)
**Number of Observations**	559	559	558	559	559	559	559	559
**Adj R-Squared**	0.062	0.072	0.059	0.074	0.076	0.077	0.067	0.071

© 2022 Hu X et al. *JAMA Network Open.*

Notes: Abbreviations: Ref. – reference. AET – adjuvant endocrine therapy.
[a] Baseline Model adjusted for sociodemographic characteristics (age at diagnosis, race, neighborhood-level education, neighborhood-level income, residence state) and clinical characteristics (cancer stage, history of chemotherapy).
[b] Model adjusted for sociodemographic characteristics and clinical characteristics in baseline model, as well as corresponding symptom cluster.

© 2022 Hu X et al. *JAMA Network Open.*

eTable 5. Multivariable Linear Regression for AET Adherence Adjusted for Psychological Symptoms Clusters

	Baseline Model[a] (1)	Baseline Model + Distress Symptoms[b] (2)	Baseline Model + Despair Symptoms[b] (3)
	Coefficient (95% CI)	Coefficient (95% CI)	Coefficient (95% CI)
**Count of symptoms with ≥ 3-point increase, during AET**		-2.6 (-4.7, -0.6)	-2.0 (-3.8, -0.2)
**Count of moderate symptoms, baseline**		-1.4 (-3.5, 0.7)	-0.6 (-2.7, 1.5)
**Race**			
Black	4.1 (-1.9, 10.1)	6.5 (0.1, 12.8)	6.8 (0.5, 13.2)
White	Ref	Ref	Ref
**Age at Diagnosis**	0.5 (0.2, 0.8)	0.5 (0.2, 0.8)	0.5 (0.3, 0.8)
**Cancer Stage**			
Stage I	Ref	Ref	Ref
Stage II	3.1 (-1.9, 8.2)	3.7 (-1.6, 9.1)	3.6 (-1.8, 8.9)
Stage III	2.0 (-6.0, 9.9)	2.0 (-6.3, 10.3)	2.2 (-6.1, 10.5)
**Neighborhood Level High School Education**	-0.1 (-0.6, 0.4)	-0.1 (-0.6, 0.4)	-0.2 (-0.7, 0.3)
**Neighborhood Level Household Income**			
<$35,000	Ref	Ref	Ref
$35,000-$44,999	-2.4 (-9.4, 4.5)	-2.9 (-10.2, 4.5)	-3.0 (-10.4, 4.3)
$45,000-$54,999	3.6 (-5.0, 12.3)	5.1 (-4.1, 14.2)	5.7 (-3.5, 14.8)
>=$55,000	3.3 (-7.5, 14.0)	4.5 (-6.8, 15.9)	5.2 (-6.2, 16.6)
**State of Residence**			
Tennessee	Ref	Ref	Ref
Arkansas	1.8 (-9.6, 13.3)	1.3 (-11.1, 13.7)	2.1 (-10.3, 14.5)
Mississippi	-2.9 (-8.5, 2.7)	-3.5 (-9.3, 2.4)	-3.2 (-9.1, 2.6)
Other	4.6 (-10.7, 20.0)	2.3 (-13.8, 18.4)	2.4 (-13.7, 18.6)
**History of chemotherapy**			
No chemotherapy	Ref	Ref	Ref
Chemotherapy within 180 days before or after AET initiation	5.1 (-1.4, 11.6)	6.1 (-0.9, 13.1)	6.2 (-0.9, 13.2)
Chemotherapy beyond 180 days before AET	-2.7 (-9.1, 3.7)	-2.3 (-8.9, 4.3)	-2.3 (-8.9, 4.3)
**Insurance type**			
Medicare Only	Ref	Ref	Ref
Medicaid or Dual	-6.0 (-13.7, 1.6)	-7.2 (-15.2, 0.8)	-7.2 (-15.3, 0.8)
**First AET type**			
Tamoxifen	Ref	Ref	Ref
Anastrozole	4.4 (-1.8, 10.6)	5.1 (-1.4, 11.6)	4.9 (-1.6, 11.5)
Exemestane	-1.3 (-10.3, 7.7)	-0.6 (-9.8, 8.7)	-0.4 (-9.7, 8.8)
Letrozole	-0.2 (-7.7, 7.2)	-0.1 (-7.9, 7.8)	-0.6 (-8.5, 7.2)
**Number of Observations**	559	510	510
**Adj R-Squared**	0.062	0.089	0.083

© 2022 Hu X et al. *JAMA Network Open.*

Notes: Abbreviations: Ref. – reference. AET – adjuvant endocrine therapy.
[a] Baseline Model adjusted for sociodemographic characteristics (age at diagnosis, race, neighborhood-level education, neighborhood-level income, residence state) and clinical characteristics (cancer stage, history of chemotherapy).
[b] Model adjusted for sociodemographic characteristics and clinical characteristics in baseline model, as well as corresponding symptom cluster.

© 2022 Hu X et al. *JAMA Network Open.*

eTable 6. Sample Characteristics of the Larger Cohort Without Requiring Continuous Insurance Enrollment or Having PCM reports

Characteristics	Black and White Patients with AET drug claims[1]			Black and White Patients with AET drug claims and continuous insurance coverage and non-missing zip codes[2]	
	Black (n=529)	White (n=1,198)		Black (n=326)	White (n=624)
**Age at Diagnosis, y**					
Mean (SD)	60.1 (12.5)	66.8 (10.5)		59.9 (13.1)	68.2 (10.7)
**Stage at Diagnosis**					
Stage I	210 (39.7)	624 (52.1)		119 (36.5)	360 (55.6)
Stage II	219 (41.4)	448 (37.4)		138 (42.3)	228 (35.2)
Stage III	100 (18.9)	126 (10.5)		69 (21.2)	59 (9.1)
**Neighborhood Level High School Education**					
Mean (SD)				78.6 (7.6)	84.0 (9.0)
**Neighborhood Level Household Income group**					
<35000				216 (66.3)	178 (27.5)
35000-44999				50 (15.3)	129 (19.9)
45000-54999				26 (8.0)	109 (16.8)
>=55000				34 (10.4)	231 (35.7)
**State**					
Tennessee				275 (84.4)	384 (59.4)
Arkansas				16 (4.9)	33 (5.1)
Mississippi				33 (10.1)	210 (32.5)
OTHER				2 (0.6)	20 (3.1)
**History of chemotherapy**					
No chemotherapy identified				175 (53.7)	460 (71.1)
Chemotherapy within 180 days before AET/after AET initiation				61 (18.7)	84 (13.0)
Chemotherapy beyond 180 days before AET				90 (27.6)	103 (15.9)
**Insurance type**					
Medicare Only				152 (46.6)	565 (87.3)
Medicaid or Dual				174 (53.4)	82 (12.7)

Notes: Abbreviations: AET – adjuvant endocrine therapy.
[1] This cohort includes Black and White patients who initiated AET (n=1,727). No requirements for having continuous Medicare and/or Medicaid coverage, non-missing zip codes, or PCM reports.
[2] This cohort includes Black and White patients who initiated AET, continuous Medicare and/or Medicaid coverage, and non-missing zip codes (n=973). No requirements for having PCM reports.

© 2022 Hu X et al. *JAMA Network Open.*
